# Comparative voxel-based dosimetry with pre-treatment Tc-99m SPECT/CT and post-treatment Y-90 PET/MRI for radioembolization with Y-90 microspheres

**DOI:** 10.1007/s12149-025-02052-5

**Published:** 2025-04-23

**Authors:** Burak Demir, Nuriye Ozlem Kucuk, Cigdem Soydal, Emre Can Celebioglu, Gizem Inal, Ecenur Dursun, Mehmet Sadık Bilgic, Digdem Kuru Oz, Atilla Halil Elhan, Kemal Metin Kir

**Affiliations:** 1 Department of Nuclear Medicine, Sanliurfa Mehmet Akif Inan Education and Research Hospital, Sanliurfa, Turkey; 2 Department of Nuclear Medicine, Ankara University Medical School, Ankara, Turkey; 3 Department of Radiology, Ankara University Medical School, Ankara, Turkey; 4 Department of Biostatistics, Ankara University Medical School, Ankara, Turkey

**Keywords:** Radioembolization, Liver, Positron-emission Tomography, Yttrium-90, Magnetic resonance imaging, Microsphere

## Abstract

**Purpose:**

The aim of this study was to investigate the differences between voxel-based dosimetry and the mean absorbed doses calculated with pre-treatment Tc-99 m-MAA SPECT/CT and post-treatment Y-90 PET/MR images. We also sought to present a detailed comparison of dose-volume histograms (DVHs) calculated from pre- and post-treatment imaging.

**Methods:**

A total of 47 treatments and 41 patients were included in the analysis as six of the treatments were retreatments of the six patients. Multicompartment and voxel-based dosimetry were performed with pre-treatment Tc-99m-MAA SPECT/CT and Y-90 PET/MRI. Correlation coefficients between the two imaging methods for the mean absorbed dose of tumor, whole liver normal tissue, perfused normal tissue, T/N ratio and tumor D10, D50 and D90 values were calculated. Additionally, differences between these values were also evaluated with Bland–Altman plots.

**Results:**

Pre-treatment Tc-99m-MAA SPECT/CT accurately predicted the dose values for healthy liver parenchyma calculated with Y-90 PET/MRI but showed lower accuracy in predicting T/N ratio and tumor doses. There were significant variations in tumor-absorbed doses for both glass and resin microspheres. Additionally, D90 values were higher when calculated with SPECT/CT than with PET/MRI, whereas D10 values were higher in PET/MRI compared to SPECT/CT.

**Conclusion:**

The findings in our study suggest that Tc-99m-MAA SPECT/CT had higher accuracy in predicting the dose to the healthy liver parenchyma compared to the tumor, maintaining its importance in treatment planning.

## Introduction

Primary liver tumors are among the most prevalent cancers worldwide [1]. Among the primary liver tumors, hepatocellular carcinoma (HCC) and intrahepatic cholangiocellular carcinoma (ICC) are the most common [2]. HCC mostly arises from a background of chronic liver disease such as alcoholic cirrhosis caused by Hepatitis B and C infection. [3]. In contrast, while chronic inflammatory diseases are predisposing factors for the development of ICC, most ICC patients have no known predisposing factors at the time of diagnosis. In addition to its primary tumors, the liver is one of the most common sites for metastasis, especially for gastrointestinal malignancies such as colorectal cancers, breast cancer, lung cancer and neuroendocrine tumors [4]. Both primary and secondary tumors of the liver have a significant impact on survival and pose important public health concerns due to their frequent occurrence in the population. For this reason, correct management and treatment of liver tumors is crucial.

While the blood supply to the normal liver parenchyma is largely provided through the portal vein, the blood supply to liver tumors is predominantly provided by the hepatic arteries [5, 6]. Through this unique vascular structure of the liver, radionuclide therapies applied via intraarterial route allow for the delivery of a high dose to the tumor while sparing the surrounding healthy liver parenchyma. In this context, transarterial radioembolization (TARE) is a treatment method based on the administration of Y-90-containing embolic microspheres through angiographic techniques via the hepatic arteries.

In treatment planning, perhaps the most important aspect is calculating the activity to be administered. Over time, different dosimetry methods have been proposed for resin (SIR-Spheres^™^; Sirtex Medical) and glass (TheraSphere^™^; Boston Scientific Corporation) microspheres. While methods such as body surface area and single compartment dosimetry were previously used for dose calculation, the superiority of multicompartment methods, which include separate dosimetric calculations for the tumor and non-tumoral segments of the liver enabling the adaptive adjustment of the desired dose, has been demonstrated in recent studies and these methods have taken their place in current guidelines [7, 8]. To perform predictive dosimetry before radioembolization, patients should undergo hepatic arteriography to assess the hepatic vascular anatomy and potential variations in vasculature. Furthermore, the dose distribution in the liver should be assessed with Tc-99m-labeled macroaggregated albumin (MAA) single-photon emission computed tomography/computed tomography (SPECT/CT) [7]. The foundation of this approach relies on the assumption that Tc-99m-labeled MAA exhibits a distribution similar to that of the microspheres used in treatment [8]. However, there are several physical differences between MAA particles and microspheres that can alter distribution of particles, such as the number of particles (significantly greater for treatment with resin microspheres [20–30 million] compared to glass [1.2–8 million]), activity per sphere (4534 Bq for glass microspheres at calibration date, 40–70 Bq for resin microspheres), size (20–30 μm for glass microspheres, 20–60 μm for resin microspheres and 10–100 μm for Tc-99m-MAA) and stability (MAA can be slowly cleared, while microspheres are permanently implanted into the vasculature) [7, 9, 10]. For these reasons, it is important to identify the differences between predicted and observed absorbed radiation doses to enable a more accurate clinical approach.

The distribution of microspheres could be assessed in vivo with post-treatment positron emission tomography (PET) imaging which utilizes rare positron emissions from the Y-90 radionuclide, enabling the absorbed dose distribution calculation for both tumor and non-tumoral tissues after treatment. In this way, the predicted absorbed doses for both tumor and non-tumor tissues in the pre-treatment phase can be verified. In dosimetry with Y-90 microspheres, most studies regarding dosimetry with Y-90 PET imaging utilize PET/CT as the imaging method [11–14]. In particular, Richetta E. et al. investigated the concordance between pre-treatment Tc-99m-MAA SPECT/CT and post-treatment PET/CT and found that the healthy parenchyma dose was better predicted compared to the tumor absorbed dose with pre-treatment imaging [12]. However, in the mentioned study there were only 10 patients treated with resin Y-90 microspheres. In a study by Gnesin S. et al., 27 treatments (7 glass, 20 resin) with Y-90 microspheres were analyzed, and the mean tumor and non-tumor absorbed radiation doses and D90 values calculated with pre-treatment Tc-99m-MAA SPECT/CT and PET/CT were compared [13]. While they observed better agreement in non-tumor absorbed doses, greater variability was observed with absorbed tumor doses. Lastly, in a study by Jadoul A. et al., it was shown that pre-treatment Tc-99m-MAA SPECT/CT can reliably predict non-tumor absorbed doses, while greater variation was observed in the mean absorbed tumor doses [15]. Apart from PET/CT, although there is limited literature on dosimetry with positron emission tomography/magnetic resonance imaging (MRI), integrated PET/MR imaging systems could also be utilized for Y-90 PET imaging. In this context, the high soft tissue contrast of MRI, combined with the simultaneous PET imaging and respiratory gating, may enable more accurate dosimetry after radioembolization.

In the dosimetric planning of radioembolization, the mean dose absorbed by the tumor and healthy liver parenchyma are generally calculated. However, another important consideration is that microspheres may not be distributed homogeneously within tumors, and due to heterogeneous dose distribution, cold spots within lesions may not absorb the radiation dose required to induce cell death. Therefore, voxel-based dosimetry methods have been proposed for both pre-treatment and post-treatment dosimetry. These methods rely on the analysis of each voxel in three-dimensional space, and it has been suggested that the dose-volume histogram values extracted from this analysis could be predictive of treatment response and play a role in calculating the necessary activity in treatment planning [16, 17].

There are a few studies investigating the reliability and predictive power of voxel-based dosimetry in pre-treatment Tc-99m-MAA SPECT/CT in radioembolization with Y-90 microspheres, and these studies usually analyzed a limited number of variables from dose-volume histograms (DVH), such as D70 and D90. In this matter, D values are the dose received by X% of the volume of interest (e.g., D70 is the dose covering 70% of the specific volume considered).

In this study, we aimed to investigate the differences between voxel-based dosimetry and the mean absorbed doses calculated with pre-treatment Tc-99m-MAA SPECT/CT and post-treatment Y-90 PET/MR images. We also sought to present a detailed comparison of dose-volume histograms (DVHs) acquired from pre- and post-treatment imaging.

## Methods

### Study design

This study is a single-center ambispective study involving patients with primary and metastatic liver tumors treated with Y-90 glass or resin microspheres and imaged with Y-90 PET/MRI in 24 h following the treatment. Patients treated between January 2021 and May 2022 were retrospectively included in this study, and patients treated between May 2022 and August 2023 were prospectively enrolled. Patients with (1) no pre-treatment Tc-99m-MAA SPECT/CT available, (2) contraindications to MRI and claustrophobia, (3) no lesion with a minimum diameter of 1 cm, (4) injection with a catheter placed in different position and angle in a treatment session and (5) multiple injections of Tc99m-MAA and microspheres were excluded from the study. This study is part of a postgraduate nuclear medicine specialist thesis and was approved by the institutional review board (Approval No. İ04–203–22). Written informed consent was obtained from all prospectively enrolled patients.

### Pre-treatment phase: hepatic artery angiography, Tc-99 m-MAA scintigraphy and treatment planning

Patients who were deemed eligible for radioembolization were evaluated with hepatic artery angiography to evaluate the vasculature. Following the intraarterial access from the femoral artery to the supplier branch of the hepatic artery of the desired segment/lobe, 150 MBq of Tc-99m MAA was injected with slow hand injection under free breathing via the micro-catheter. All invasive angiographic procedures (digital subtraction angiography [DSA] and cone-beam CT) were performed by two interventional radiologists with more than 10 years of experience. Following the angiography session, planar scintigraphy of the thoraco-abdominal region is performed to evaluate pulmonary and gastrointestinal shunt, along with a tomographic SPECT/CT acquisition to assess the distribution of Tc-99m activity, which is then used for dosimetric treatment planning calculations. Approximately 1–2 h after the angiographic procedure, planar scintigraphy and SPECT/CT imaging was performed. For Tc-99m-MAA SPECT/CT and planar imaging, GE Healthcare Optima NM/CT 640 and NM/CT 860 SPECT/CT (GE Healthcare, Milwaukee, Wisconsin, USA) systems were utilized. The parameters used for SPECT/CT acquisition included low-energy high resolution collimator, 90 frames with 20 s each, a 128 × 128 matrix, a 10% symmetric window around the 140 keV energy peak, and scatter correction with an energy window of 100–125 keV. In addition, a non-circular 360° angle with body-contouring was utilized and resulting voxel size was 4.42 × 4.42 × 2.50 mm. The SPECT images were accompanied by low-dose helical non-contrast CT acquisition with 120 kV, 30 mAs with 2.5 mm slice thickness for measured attenuation correction (AC) and localization. For reconstruction, Ordered Subset Expectation Maximization (OSEM) algorithm with 4 iterations and 10 subsets was utilized. Additionally, resolution recovery with the vendor-provided application and a post-processing Butterworth filter with a cutoff frequency of 0.4 cycles/cm was applied.

Treatments were planned with a multicompartmental dosimetry model using Simplicit90Y™ (Mirada Medical, US) in accordance with current guidelines [7, 8]. The radiation dose absorbed by the lungs was calculated with the lung shunt fraction calculated with pre-treatment Tc-99m-MAA planar scintigraphy. The 30 Gy lung tissue absorbed dose limit per treatment and 50 Gy of cumulative dose limits were utilized. Tc-99m-MAA SPECT/CT was registered with manual rigid registration with the existing previous diagnostic contrast-enhanced CT or MRI of the patient before tumor and liver segmentation. For the dose calculations, the local dose deposition method and patient-relative conversion were utilized. The minimum tumor dose of 200 Gy for the metastases and 250 Gy for hepatocellular carcinoma was targeted if possible and whole liver normal tissue dose was limited to 50–90 Gy for glass microspheres. For the resin microspheres, the minimum tumor dose of 100 Gy was targeted and perfused liver normal tissue dose was limited to 40 Gy. The lung shunt fraction was calculated using the geometric means of the lung and liver regions-of-interests (ROIs) on anterior and posterior planar images. In cases of the effective treatment exceeding the lung dose limits, the treatment was not performed, and the clinician of the patient was informed. Tc-99m-MAA SPECT/CT sequences were used for predicting the dose absorbed by tumor and non-tumor liver tissues. Within two weeks, radioembolization procedures were performed.

### Therapeutic phase: radioembolization, Y-90 PET/MRI protocol and dosimetric verification

For the radioembolization, the microspheres were slowly administered over 5–10 min in combination with 5% dextrose solution to prevent vasospasm. The same position and angle of the infusion catheter were maintained, when possible, as in the simulation using Tc-99m MAA. In the case of injection from different positions and angles (e.g., to prevent gastric reflux, to preserve liver tissue or to treat a wider region) the patient was excluded from the study. Patients were then monitored overnight in the clinic, followed by Y-90 PET/MR imaging conducted within 24 h post-procedure.

Y-90 PET/MR imaging was performed with an integrated GE Healthcare SIGNA^™^ PET/MR (GE Healthcare, Milwaukee, Wisconsin, USA) system with a 3T MRI component. Depending on the size of the liver, one-bed position with 30 min of acquisition time or two-bed positions with 20 min per bed position centered on the liver were used for PET imaging. No contrast medium was administered for MR imaging. High-resolution non-contrast coronal SS-FSE, axial T2-weighted (T2w) PROPELLER and T2wFS-PROPELLER, axial 3D T1w-LAVA, diffusion-weighted imaging with b-values of 50, 800, 1000, and 2000, and apparent diffusion coefficient map sequences were acquired. For PET acquisition, reconstruction was performed with the VPFX algorithm using ordered-subset expectation maximization (OSEM) with 1 iteration and 16 subsets. The matrix size was 256 × 256 with a resulting voxel size of 2.34 × 2.34 × 2.78 mm. Attenuation correction was applied using maps generated with MRAC sequences. Respiratory gating and time-of-flight were enabled, and a post-processing Gaussian filter (5.0 mm at full width at half maximum) was employed.

The radiation dose absorbed by the lungs was calculated with the lung shunt fraction calculated with pre-treatment Tc-99m-MAA planar scintigraphy. The whole liver, tumor, and non-tumoral liver compartments were manually delineated on 2.2 mm thin slice T1-weighted LAVA sequences and verified using T2-weighted PROPELLER sequences by a nuclear medicine specialist experienced in PET/MRI. In addition, perfused volumes were delineated manually based on activity on SPECT/PET images. As almost all patients were treated with unilobar treatments, the perfused segment limits determined upon inspecting SPECT/PET and MRI images,the perfused and non-perfused segments of the liver were split with linear lines. Since the PET and MR images were inherently aligned, no additional registration was needed. The pre-treatment Tc-99m-MAA SPECT/CT images were registered to MRI sequences of the Y-90 PET/MRI acquisition with a manual rigid registration. Subsequently, the same segmentations were also used for the calculation with the SPECT images.

Post-therapy dosimetry with Y-90 PET/MRI was also performed with Simplicit90Y. Simplicit90Y utilizes a patient-relative conversion method, and this method was used after subtraction of previously measured residual activity in vial and catheter. To convert PET images to the radiation dose maps, local deposition method was utilized. Voxel-wise conversion of PET and SPECT images to Gy dose maps was performed with the following formula:

$$Activity \left[GBq\right]= \frac{D\left[Gy\right]\times Mass\left[kg\right]}{49.67 J/GBq}$$

**Fig. 1:**
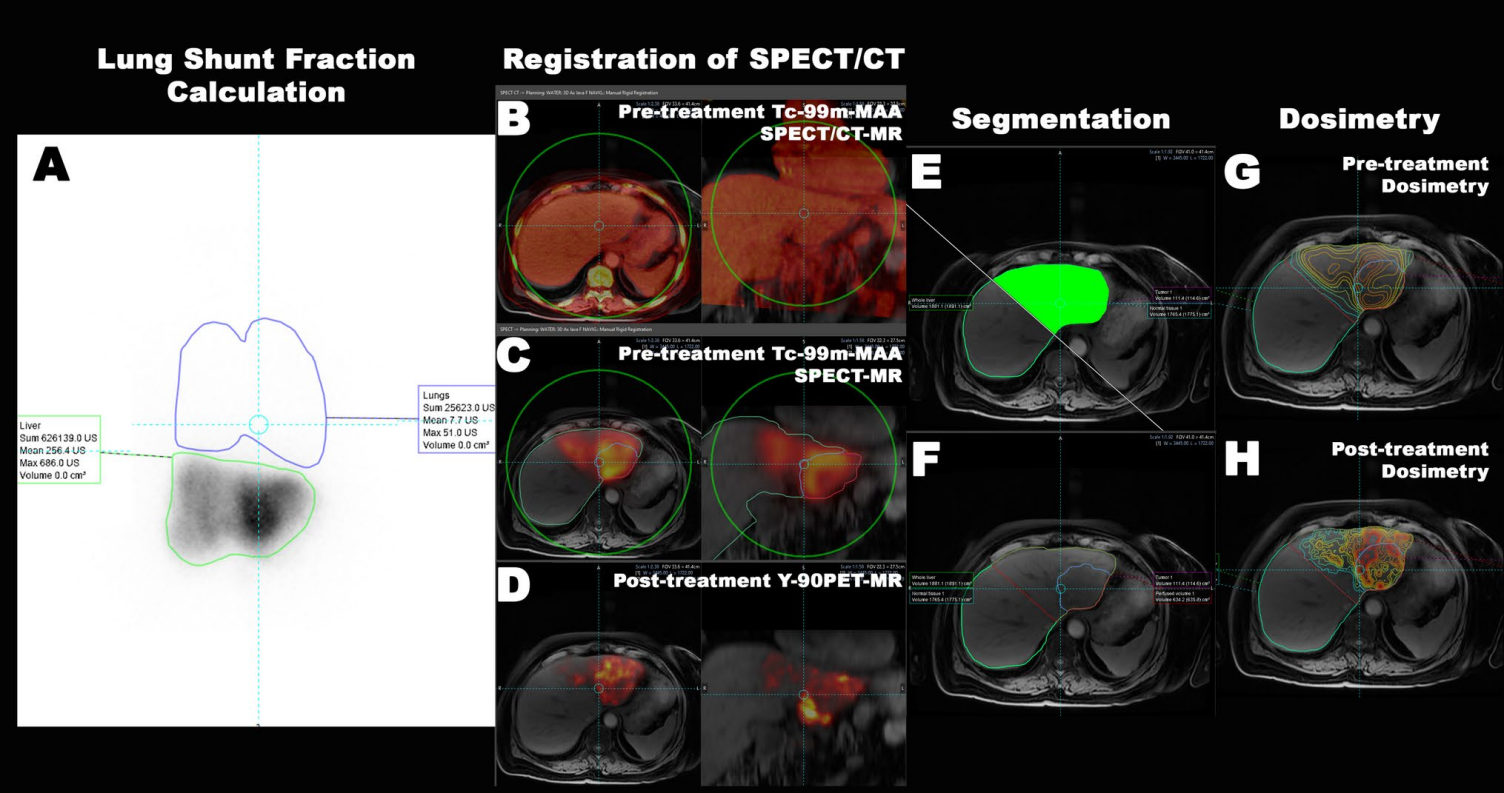
The summary of the workflow is given in the figure. First, the lung shunt fraction was calculated with the anterior–posterior planar Tc-99m-MAA scintigraphy images. Secondly, the pre-treatment SPECT/CT images were registered to the Y-90 PET/MRI. As the PET images are inherently aligned with the MRI, no further registration was needed. Later, the whole liver, perfused liver, tumor, normal tissue and perfused normal tissue compartments were segmented. Lastly, dosimetry with the Tc-99m-MAA SPECT and Y-90 PET images was performed with the same segmentations

Mean absorbed radiation dose values in Gy were calculated for every delineated compartment for both Tc-99m-MAA SPECT/CT and Y-90 PET/MRI. Furthermore, tumor-to-normal tissue (T/N) values were calculated by dividing the tumor-absorbed dose by the absorbed dose in perfused normal tissue. Additionally, dose‒volume histograms were created by quantifying the absorbed radiation dose of each voxel in the determined compartment and sorting these voxels according to their values. A summary of the workflow is given in Fig. 1**.**

### Statistical analysis

Descriptive statistics are summarized as counts and percentages for categorical variables; means, standard deviations and medians (minimum–maximum) for others. For statistical analysis, p values smaller than 0.05 were considered to be statistically significant.

Tc-99m-MAA SPECT/CT and Y-90 PET/MRI-derived data were compared for the predicted and actual mean doses, and intraclass correlation coefficients (ICC) were investigated via reliability analysis. ICC values smaller than 0.5 were considered as poor agreement, the values between 0.5 and 0.75 as moderate agreement, 0.75–0.9 as good agreement and 0.9–1.0 as excellent agreement [18]. Furthermore, the relationships between the variables calculated with Tc-99m-MAA SPECT/CT and Y-90 PET/MRI were compared and visualized with linear regression analyses and R^2^ values were calculated. As most of the variables had non-normal distribution, the Wilcoxon signed-rank test was employed for paired samples to detect differences in measurements between tests. Statistical analyses were performed using IBM SPSS Version 27.0. Furthermore, Bland–Altman plots generated with the BA-plotteR tool were utilized to investigate differences and limits of agreement [19]. For visualization of SPECT and PET/MRI data, Simplicit90Y and 3D Slicer version 5.2 were utilized [20].

## Results

### Patient population and descriptive data

**Fig. 2:**
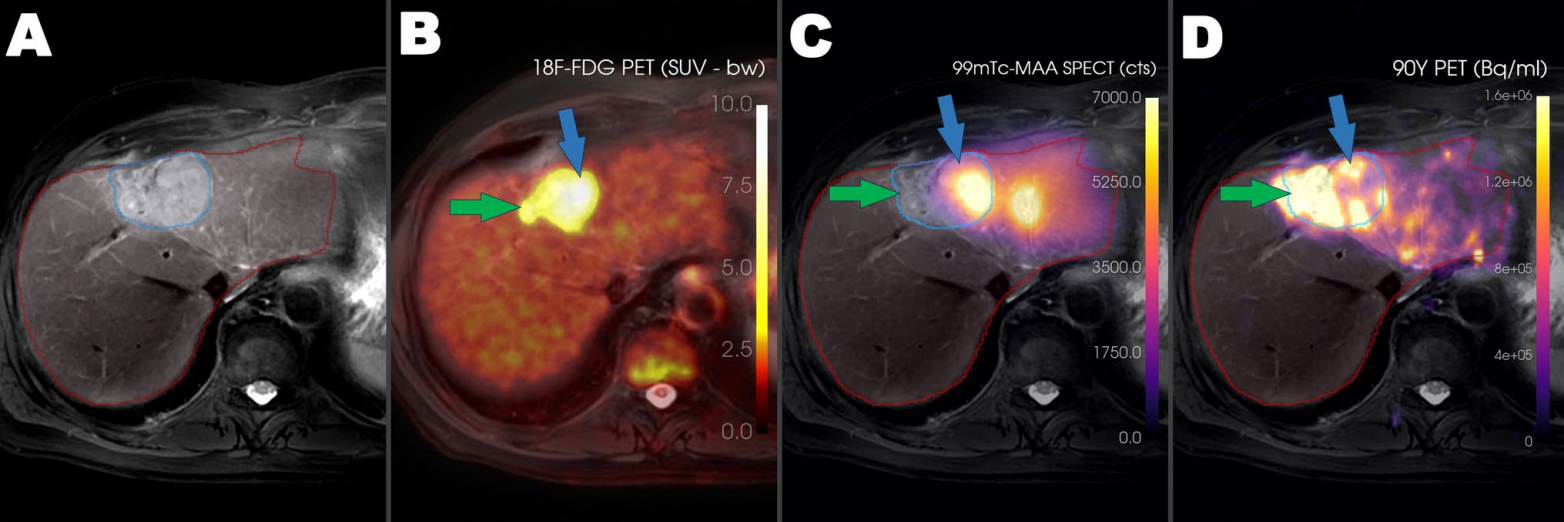
A 66-year-old man was treated for solitary metastasis of colorectal carcinoma. The lesion located in segment II-IVA of the liver was visualized on axial T2-weighted MR (**A**) and axial PET/MR fusion (**B**) images. Pre-treatment Tc-99m-MAA SPECT-MRI fusion images revealed intense uptake of Tc-99m-MAA in the segment II component of the lesion (**C**, blue arrow), while the non-perfused part of the tumor was also identified (**C**, green arrow). An additional dose was administered during treatment to ensure complete coverage of the tumor and could be visualized in post-treatment Y-90 PET/MR images (**D**). Consequently, the patient was excluded from the study.

The pre- and post-treatment data of 94 treatments were evaluated. After excluding patients with the previously mentioned criteria, 47 treatments and 41 patients were included in the analysis. Six of the included treatments were the re-treatments of the patients already included in the study. Among the treatments, 27 treatments were performed with glass, and 20 treatments were performed with resin microspheres. The reasons for exclusion were as follows: 7 cases due to inaccessible Tc-99m-MAA SPECT/CT images, 7 cases where the injection was administered from a more proximal position during treatment, 21 cases with more selective injection in treatment, and 12 cases where additional injections were administered during therapy to cover more tumor tissue. Example images of an excluded patient is given in Fig. 2**.**

Of those patients, 24 were male and 17 were female. The median age of the patients was 63 years (range: 38–82). Most patients had liver tumors originating from colorectal carcinoma (20, 49%) and hepatocellular carcinoma (11, 27%), while the rest were diagnosed with breast, cholangiocellular, and adrenocortical carcinomas, neuroendocrine tumors and uveal melanoma. Most of the patients (71%) presented with bilobar disease. Of those patients, 29 (71%) had bilobar disease, 9 (22%) had tumors confined to the right lobe of the liver, and 3 (7%) had tumors confined to the left lobe. Thirty-five (74%) treatments were planned as unilobar treatments to the right lobe, 11 (23%) as unilobar treatments to the left lobe, and 1 (2%) patient received segmental treatment. Descriptive data of the patient population are given in Table 1**.**

**Table 1:** Descriptive data of the patients included in the study

Descriptive data of the patient population	
Gender and Age	
Male	24 (58%)
Female	17 (42%)
Median Age	63 years (range: 38–82)
Previous Treatments	
Chemotherapy	24 (58%)
Tyrosine kinase/VEGF inhibitors	9 (22%)
TARE/TACE	13 (32%)
Peptide Receptor Radionuclide Treatment	1 (2%)
Presence of Cirrhosis	
Present	14 (34%)
None	27 (66%)
Child Pugh Score	
A5	35 (85%)
A6	2 (5%)
B7	3 (7%)
B8	1 (2%)
ALBI Grade	
Grade 1	33 (80%)
Grade 2	8 (20%)
Extrahepatic Metastasis	
Present	23 (56%)
None	18 (44%)
Tumor Distribution in the Liver	
Bilobar	29 (71%)
Only Right Lobe	9 (22%)
Only Left Lobe	3 (7%)
Volumetric Variables	
Liver Volume (median)	1700 mL (range: 941–3033)
Tumor Volume (median)	116.2 mL (range: 8–1148)
Perfused Volume (median)	1063.2 mL (range: 248.7–2408)
Perfused Tumor Volume (median)	100.9 mL (range: 4.9–718.2)
Perfused Healthy Liver Volume (median)	960.7 mL (range: 128.6–2096.3)

The median activity delivered to the perfused volume was 2.35 GBq (range: 1.20–4.73 GBq) for glass microspheres and 1.20 GBq (range: 0.49–2.40 GBq) for resin microspheres. For glass microspheres, all treatments were applied in the third or fourth days of the first week after the calibration date. In addition, all resin microsphere treatments were applied one or two days after the calibration date.

### Comparison of the mean absorbed radiation doses calculated with Tc-99m-MAA SPECT/CT and Y-90 PET/MRI

For patients treated with glass microspheres, the median mean tumor absorbed dose calculated with Y-90 PET/MRI was 207.60 Gy (range: 52.5–636.7 Gy), while the median value predicted with Tc-99m-MAA SPECT/CT was 208.42 Gy (range: 76.2–407.9 Gy). For the resin microspheres, the corresponding values were 113.3 Gy (range: 56.2–314.1 Gy) for Y-90 PET/MRI and 102.50 Gy (range: 45.0–508.9 Gy) for Tc-99m-MAA SPECT/CT.

The median whole liver normal tissue mean absorbed dose was 55.0 Gy (range: 6.0–84.0 Gy) for Y-90 PET/MRI and 52.9 Gy (range: 8.1–84.5 Gy) for Tc-99m-MAA SPECT/CT in the patients treated with glass microspheres. For the resin microspheres, the corresponding values were 20.6 Gy (range: 3.7–42.5 Gy) for Y-90 PET/MRI and 20.55 Gy (range: 9.3–43.1 Gy) for Tc-99m-MAA SPECT/CT.

**Fig. 3:**
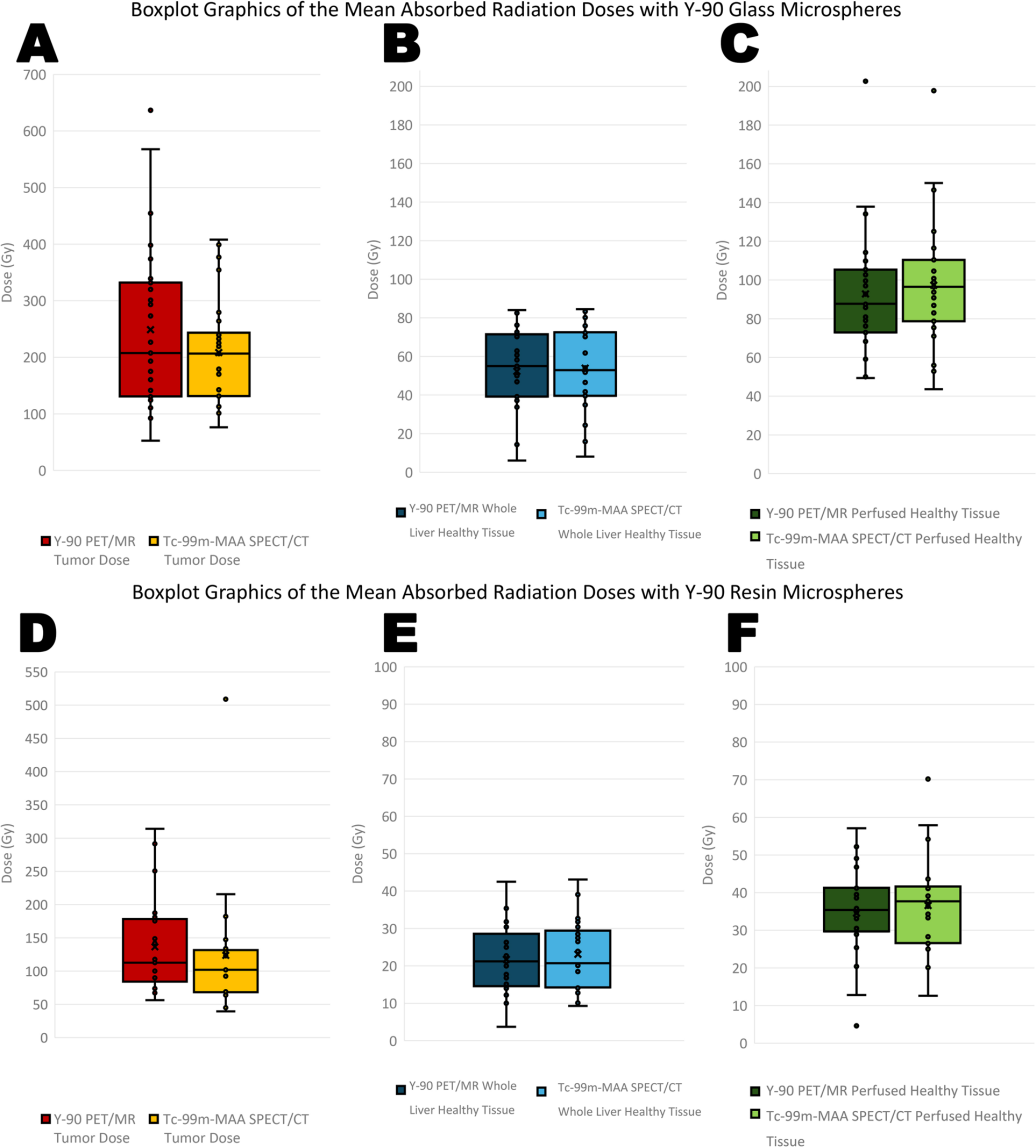
The boxplot graphics of the mean absorbed radiation doses by the tumor (**A**, **D**), whole liver health tissue (**B**, **E**) and perfused healthy tissue (**C**, **F**) for glass and resin microspheres are given

For perfused normal liver tissue doses, the median dose values calculated with Y-90 PET/MRI and Tc-99m-MAA SPECT/CT for glass microspheres were 87.7 Gy (range: 49.4–202.7 Gy) and 96.5 Gy (range: 43.6–197.8 Gy), respectively. For the resin microspheres, the corresponding values were 35.1 Gy (range: 4.6–57.1 Gy) and 37.05 Gy (range: 12.6–70.2 Gy). The boxplot graphics of the calculated doses for tumor, whole liver and perfused healthy liver are given in the Fig. 3**.**

Lastly, median T/N ratios calculated with Y-90 PET/MRI and Tc-99m-MAA SPECT/CT were 2.32 (0.61–9.2) and 1.87 (1.08–7.30) for glass microspheres, 3.75 (1.16–22.8) and 3.00 (1.17–20.4) for resin microspheres, respectively.

In matched samples for glass microspheres, Wilcoxon signed-rank tests did not reveal any statistically significant difference for perfused normal liver tissue dose (*p* = 0.058), and whole liver normal tissue dose (*p* = 0.061) calculated with the two imaging modalities. Nevertheless, the analysis of T/N ratios (*p* = 0.019) and the mean absorbed tumor dose (*p* = 0.031) calculated with Tc-99m-MAA SPECT/CT and Y-90 PET/MRI for glass microspheres revealed a statistically significant difference between the two imaging modalities. For resin microspheres, Wilcoxon signed-rank tests revealed no statistically significant differences between the mean absorbed tumor dose (*p* = 0.108), perfused normal liver tissue dose (*p* = 0.126), whole liver normal tissue dose (*p* = 0.059), and T/N ratio (*p* = 0.108) calculated with Tc-99m-MAA SPECT/CT and Y-90 PET/MRI.

**Fig. 4:**
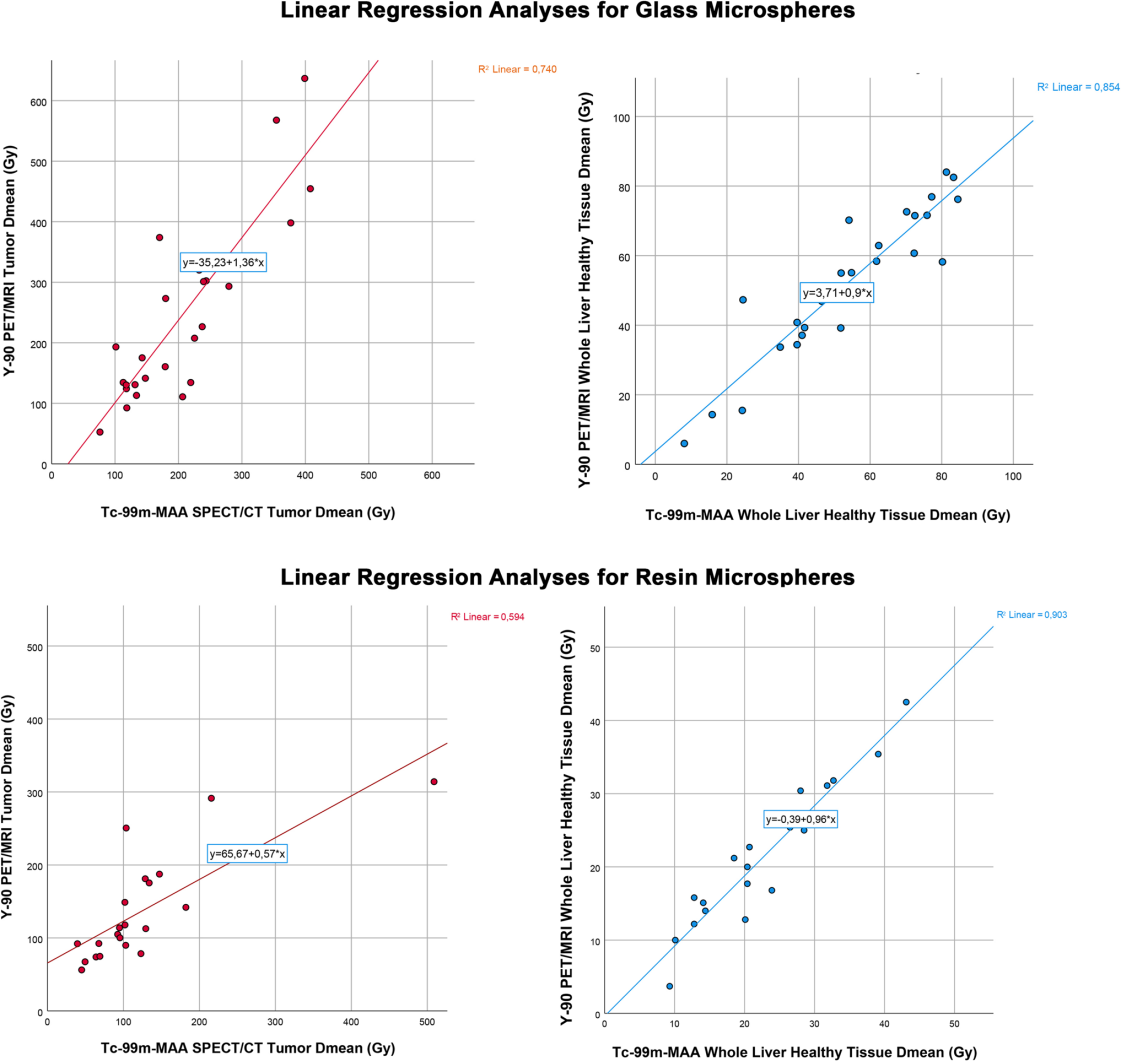
The scatter plots of the mean absorbed radiation doses of the tumor and whole liver healthy tissue compartments are given in the figure. The R^2^ values calculated for the healthy tissues were significantly higher than those for the tumors

To investigate the agreement between dose metrics calculated with pre-treatment Tc-99m-MAA SPECT/CT and Y-90 PET/MRI, a two-way mixed-effects model was utilized to calculate ICCs. In this analysis, the ICC value for the Tc-99m-MAA SPECT/CT and Y-90 PET/MRI-derived mean tumor absorbed doses was 0.776 (p < 0.001) for the glass microspheres and 0.735 (p < 0.001) for the resin microspheres. For perfused normal liver tissue absorbed doses, the ICC value was 0.879 (*p* < 0.001) for glass microspheres and 0.904 (*p* < 0.001) for resin microspheres. Additionally, for the whole liver normal tissue absorbed doses, the ICC value was 0.924 (*p* < 0.001) for glass microspheres and 0.948 (*p* < 0.001) for resin microspheres. Finally, for the T/N ratios, the ICC value was 0.726 (*p* < 0.001) for the glass microspheres and 0.509 (*p* = 0.009) for the resin microspheres. The scatter plot graphics and the linear regression lines with R^2^ values of the tumor and normal tissue compartments for both microspheres are given in Fig. 4.

**Fig. 5:**
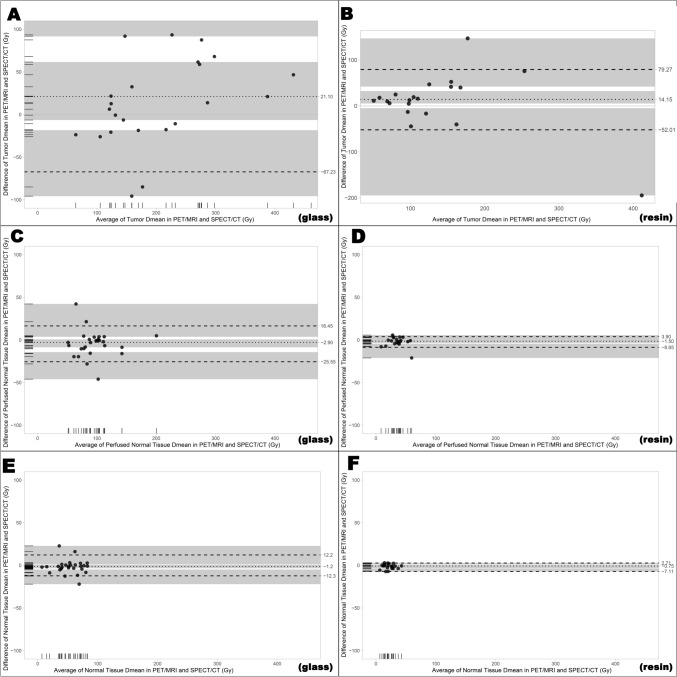
Bland–Altman plots of mean absorbed tumor, perfused and whole liver normal tissue doses calculated from Y-90 PET/MRI and Tc-99m-MAA SPECT/CT for glass (**A**, **C**, **E**) and resin (**B**, **D**, **F**) microspheres

Furthermore, to investigate the limits of agreement, Bland–Altman plots were utilized. For glass microspheres, difference between Y-90 PET/MRI and Tc-99m-MAA SPECT/CT calculated mean absorbed dose was calculated as 21.1 Gy (95% CI: −6.4/61.6) and limits of agreement were 210.49 Gy (upper) and −67.23 Gy (lower). Additionally, differences of perfused normal tissue dose and whole liver normal tissue dose calculated with Y-90 PET/MRI and Tc-99m-MAA SPECT/CT were −2.9 Gy (95% CI: −8.6/0.6) and −1.2 Gy (95% CI: −3.4/0.3), respectively. Limits of agreement were calculated as 16.45 Gy and −25.55 Gy for perfused normal liver tissue dose and as 12.2 Gy and −12.3 Gy for whole liver normal tissue dose (Fig. 5).

For resin microspheres, the difference between Y-90 PET/MRI and Tc-99m-MAA SPECT/CT-based mean absorbed dose of tumors was calculated as 14.15 Gy (95% CI: 5.3/32.4) and limits of agreement were 79.27 Gy (upper) and −52.01 Gy (lower). Additionally, differences of perfused normal tissue dose and whole liver normal tissue dose calculated with Y-90 PET/MRI and Tc-99m-MAA SPECT/CT were −1.5 Gy (95% CI: −3.5/0.1) and −0.75 Gy (95% CI: −3.1/−0.4), respectively. Limits of agreement were calculated as 3.9 Gy and −8.65 Gy for perfused normal liver tissue dose and as 2.71 Gy and −7.11 Gy for whole liver normal tissue dose **(**Fig. 5**).**

### Comparison of the dose-volume histograms calculated with Tc-99 m-MAA SPECT/CT and Y-90 PET/MRI

**Fig. 6:**
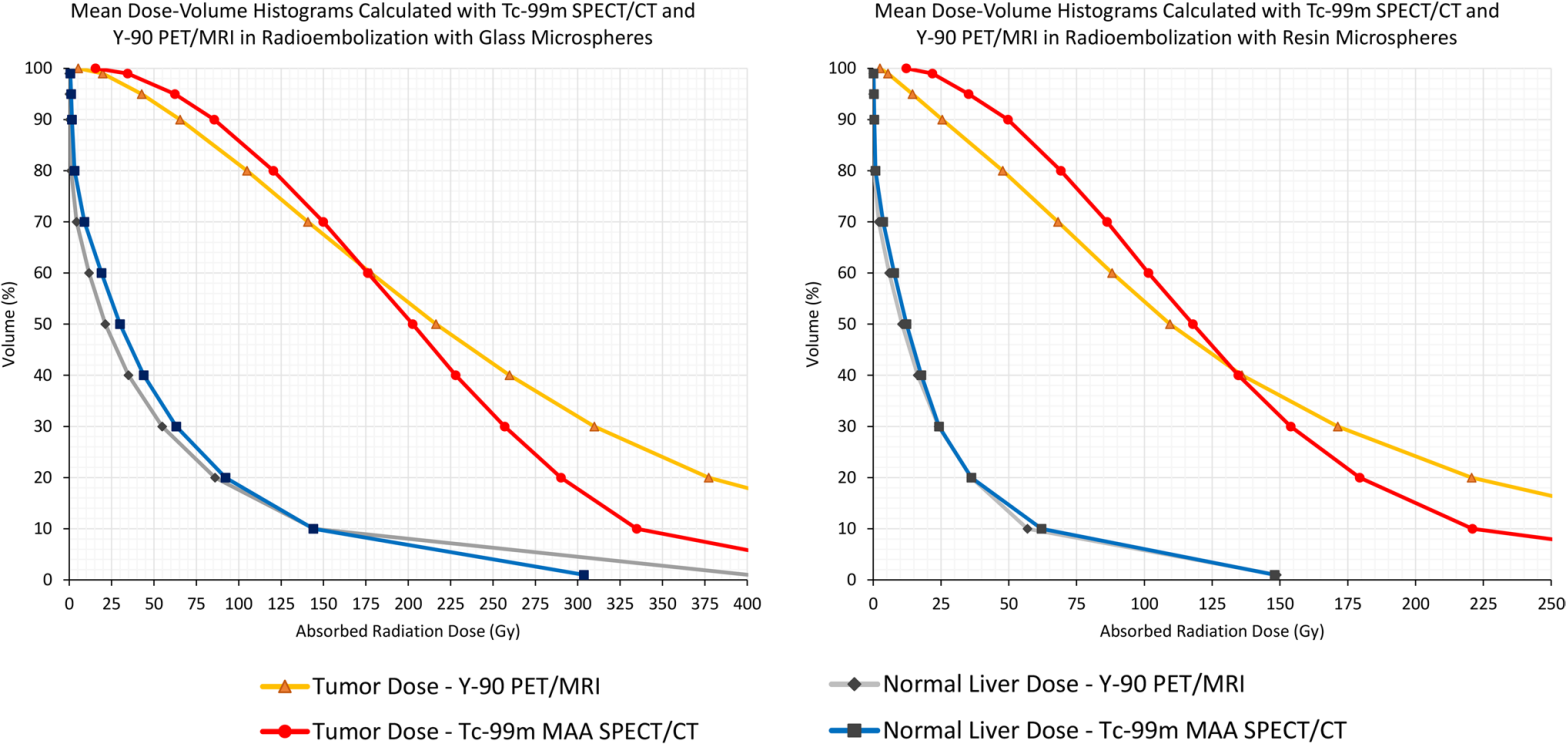
Mean dose‒volume histograms for tumor and whole liver normal tissue calculated with Tc-99m-MAA SPECT/CT and Y-90 PET/MRI after radioembolization with glass and resin microspheres

Dose‒volume histograms were calculated for the absorbed doses in tumors. In addition to the average dose values, the correlations between dose-volume histogram-derived variables were also investigated for the two imaging modalities. ICC values for the tumor tissue D10, D50 and D90 values for glass microspheres were 0.687 (*p* < 0.001), 0.792 (*p* < 0.001) and 0.837 (*p* = 0.002), respectively. The corresponding values for resin microspheres were 0.735 (*p* < 0.001), 0.571 (*p* = 0.003) and 0.614 (*p* = 0.002), respectively. Additionally, averaged dose-volume histograms for tumors and whole liver normal tissue calculated with both imaging methods are also included in Fig. 6**.**

**Fig. 7:**
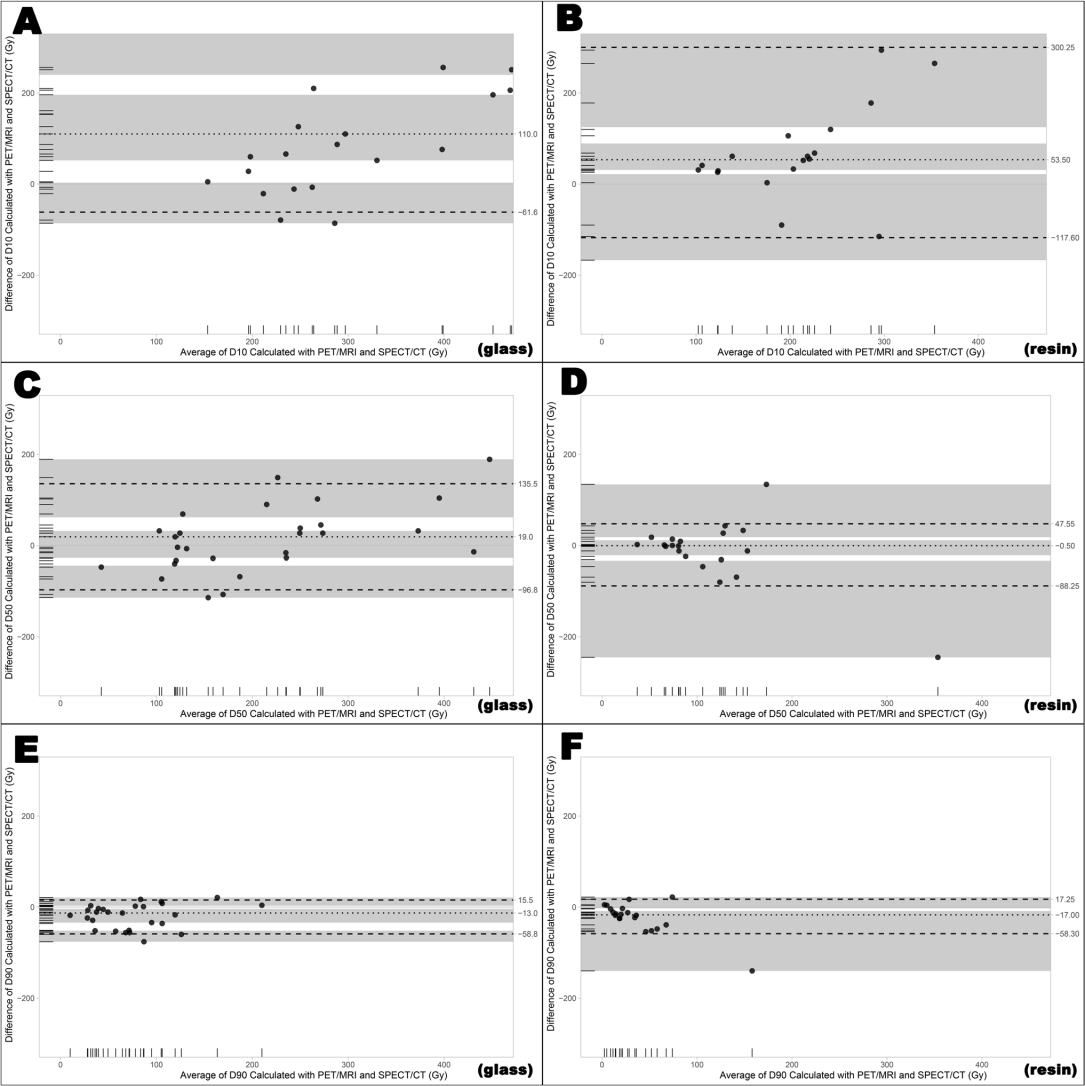
Bland–Altman plots for tumor D10, D50, D90 values calculated with Y-90 PET/MRI and Tc-99m-MAA SPECT/CT for glass (**A**, **C**, **E**) and resin (**B**, **D**, **F**) microspheres

For glass microspheres, the medians of D10, D50, and D90 values for tumor absorbed radiation doses calculated with Tc-99m-MAA SPECT/CT and Y-90 PET/MRI -representing low, medium, and high D values- were 277 Gy (range: 151–782) and 369 Gy (range: 156–1493) for D10, 211 Gy (range: 66–438) and 162 Gy (range: 19–542) for D50, and 84 Gy (range: 19–208) and 46 Gy (range: 1–212) for D90. While statistically significant differences were observed between the values calculated with both methods for D10 (*p* < 0.001) and D90 (*p* < 0.001), no significant difference was found for D50 (*p* = 0.478). In addition, Bland–Altman plots for glass and resin microspheres are given in Fig. 7**.** Difference between Y-90 PET/MRI and Tc-99m-MAA SPECT/CT calculated D10, D50 and D90 values were found as 110 Gy (95% CI: 60–196), 19 Gy (95% CI: [− 27]–32) and −13 Gy (95% CI: [−34]–[−3]), respectively. Limits of agreement were 537.8 Gy (upper) and −61.6 Gy (lower) for D10, 135.5 Gy (upper) and −96.8 Gy (lower) for D50 and 15.5 Gy (upper) and −58.8 Gy (lower) for D90.

For the resin microspheres, the medians of D10, D50, and D90 values for the tumor absorbed radiation doses calculated with Tc-99m-MAA SPECT/CT and Y-90 PET/MRI were as follows: 188 Gy (range: 86–858) and 244 Gy (range: 118–890) for D10, 103 Gy (range: 36–476) and 84 Gy (range: 38–240) for D50, and 31 Gy (range: 0–228) and 20 Gy (range: 5–88) for D90. The same analysis was performed for resin microspheres, and similarly, statistically significant differences were found for the D10 (*p* = 0.019) and D90 (*p* = 0.004) values, while no statistically significant difference was observed for the D50 (*p* = 0.629) values between the two imaging methods. Differences between Y-90 PET/MRI and Tc-99m-MAA SPECT/CT D10, D50 and D90 values were found as 53.5 Gy (95% CI: 30–50), −0.5 Gy (95% CI: [−22.81]–12.81) and −17 Gy (95% CI: [−25]–[−7.5]), respectively. Limits of agreement were 300.25 Gy (upper) and −117.6 Gy (lower) for D10, 47.55 Gy (upper) and −88.25 Gy (lower) for D50, and 17.25 Gy (upper) and −58.3 Gy (lower) for D90 (Fig. 7).

**Fig. 8:**
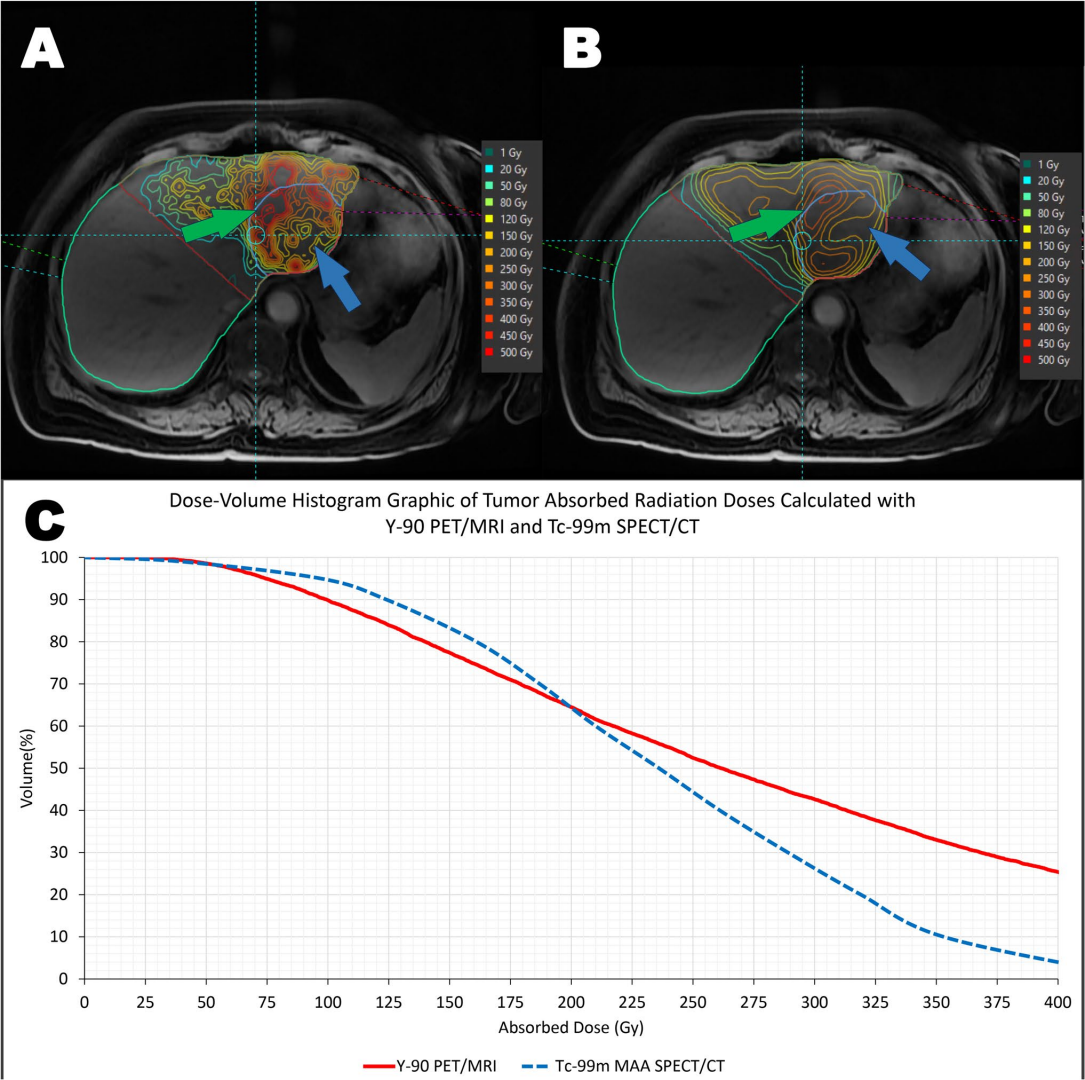
A 76-year-old woman who was diagnosed with metastatic rectal carcinoma and treated with 2.17 GBq glass microspheres. Comparative voxel-based dosimetry was performed using post-treatment Y-90 PET/MR (**A**) and pre-treatment Tc-99m-MAA SPECT/CT-MRI (**B**) images. The predicted mean dose was 240 Gy, while the mean absorbed dose calculated from post-treatment imaging was 301 Gy. Notably, significant differences were observed in the dose‒volume histograms (**C**) generated with both methods. Y-90 PET/MRI exhibited lower D80 and D90 values than Tc-99m-MAA SPECT/CT, likely due to a larger hypoperfused segment visualized on Y-90 PET/MRI relative to Tc-99m-MAA SPECT/CT (blue arrows). On the other hand, Y-90 PET/MRI had higher D10-40 values than Tc-99m-MAA SPECT/CT, which can be attributed to the presence of ‘hot spots’ observed in Y-90 PET/MRI compared to Tc-99m-MAA SPECT/CT (green arrows)

For both types of microspheres, D10 values calculated with Tc-99m-MAA SPECT/CT were lower than those calculated with Y-90 PET/MRI, while D90 values calculated with Y-90 PET/MRI were lower than those calculated with Tc-99m-MAA SPECT/CT (Fig. 8).

## Discussion

**Fig. 9:**
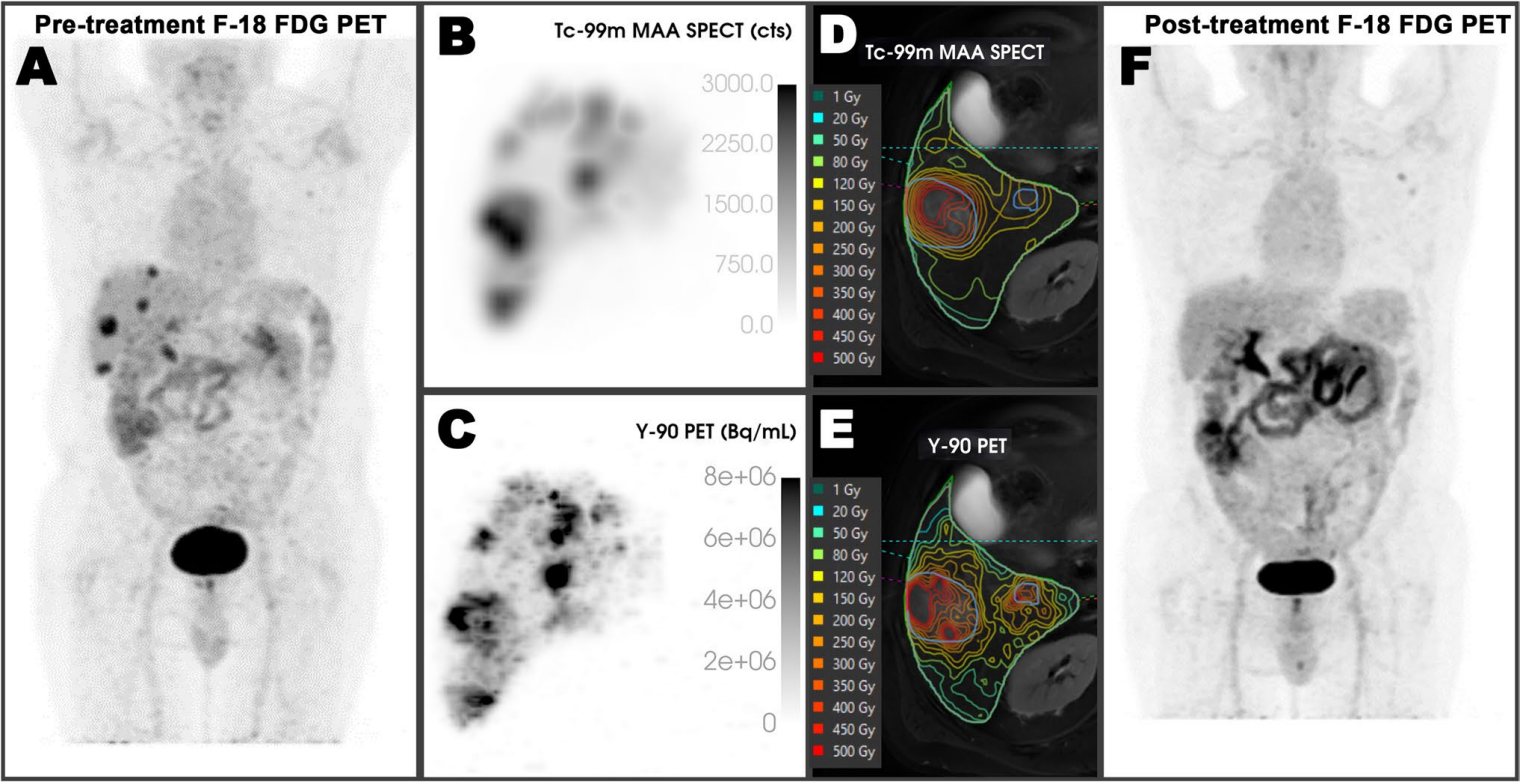
**A** 67-year-old man underwent treatment for multiple metastases of colorectal carcinoma in the liver with 2.80 GBq Y-90 glass microspheres. Intense accumulation of Tc-99m-MAA in the tumors was observed in both pre-treatment SPECT MIP images (**B**) and Tc-99m-MAA SPECT/CT -MRI fusion images (**D**). Similarly, post-treatment PET MIP (**C**) and Y-90 PET/MRI fusion images (**E**) also revealed intense uptake of Y-90 microspheres in the tumors. The mean radiation dose to tumors was calculated as 179.80 Gy with pre-treatment SPECT and 273.2 Gy with post-treatment PET. On follow-up ^18^F-FDG PET imaging complete response was observed (**F**)

In this study, pre-treatment Tc-99m-MAA SPECT/CT images were compared with Y-90 PET/MR images to evaluate their difference and concordance rates. The mean doses calculated for whole liver normal tissue and perfused normal tissue from pre- and post-treatment images did not significantly differ from each other for glass and resin microspheres. However, significant differences were observed between the T/N ratio and mean tumor doses calculated with the Tc-99m-MAA SPECT/CT and Y-90 PET/MR images for glass microspheres. These findings could be attributed to the higher resolution of PET imaging compared to SPECT imaging, resulting in better recovery of contrast between the tumor and normal tissue especially for smaller lesions (Fig. 9). Additionally, the use of low-dose CT in Tc-99m-MAA SPECT/CT images which necessitates the usage of another diagnostic imaging and co-registration might have affected the dosimetry. However, Y-90 PET/MRI with integrated MR sequences may have enabled more accurate segmentation. Although manual registration was applied for every treatment, variations due to both patient-related factors (such as respiratory artifacts and patient positioning) and technical factors (such as lack of respiratory correction, absence of contrast injection, and inaccurate registration) may have also contributed to these differences in the results. In addition, another factor that could have affected the results is the differences in the shape, size, and number of particles of the microspheres used in treatment compared to macroaggregated albumin.

To test the concordance between pre-treatment and post-treatment dosimetry with Tc-99m-MAA SPECT/CT and Y-90 PET/MRI, reliability analyses were performed. In this regard, for the whole liver and perfused normal tissue compartments, the correlation coefficient between Y-90 PET/MRI and Tc-99m-MAA SPECT/CT was greater than that of the ICCs for the tumor dose and T/N. As a result, while Tc-99m-MAA SPECT/CT can predict tumor and T/N values up to a certain point, it can accurately predict normal tissue doses. The relatively small volume of the tumors compared to that of normal liver tissue, along with the distribution of administered activity essentially into two compartments, may have contributed to these findings. In this setting, transferring a certain amount of activity from the normal tissue compartment to the tumoral compartment, or vice versa, may not significantly alter the dose in normal tissue, but it can substantially affect the absorbed doses by the tumor tissue. This could potentially lead to better prediction of normal tissue doses while also leading to relatively inaccurate prediction of tumor doses. Other factors that could potentially reduce the agreement might include technical aspects such as catheter position, angle, injection rate, and the possibility of vasospasm during injection.

Dose-volume histograms are usually utilized in external beam radiotherapies in routine practice and the mean radiation dose values are usually considered in the radioembolization treatments. However, dose-volume histograms might be useful in evaluating the heterogeneity of the distribution of microspheres. In addition to the DVHs, there are several effective dose metrics that take into account the effect of radiation on different tissues, such as biological effective dose (BED) and equivalent uniform dose (EUD), and these metrics also may be used for further prediction and adjustment of radioembolization treatments. Furthermore, these mentioned variables have been shown to be useful in the prediction of the response [21, 22].

In this study, we have analyzed the differences in the DVHs calculated with the Tc-99m-MAA SPECT and Y-90 PET. In the dose-volume histograms calculated for the absorbed radiation dose by the tumor tissue using both imaging modalities, lower D10 values were calculated with Tc-99m-MAA SPECT/CT, while D90 values were calculated lower with Y-90 PET/MRI. No statistically significant difference was observed for the D50 value. If the possible reasons for this situation are investigated, it can be suggested that small regions with low activity may not be clearly imaged due to the lower spatial resolution and contrast of SPECT compared to that of PET, resulting in higher D90 values with Tc-99m-MAA SPECT/CT. On the other hand, higher values of D10 with Y-90 PET/MRI could be attributed to the presence of hot spots that may arise during reconstruction due to the low signal-to-noise ratios in Y-90 PET imaging; however, microscopic heterogeneities of the microspheres might also have contributed to this result.

In this matter, in a study by Rhee et al., they compared pre-therapy Tc-99m-SPECT and post-therapy Y-90 PET/CT images and found a positive correlation between T/N values calculated with the two imaging modalities (rho = 0.648) [23]. Furthermore, in the same study, the T/N ratios predicted by Tc-99m-SPECT were lower than those calculated by PET imaging, similar to the findings in our study. Also, in a study by Jadoul A. et al., Tc-99m-SPECT and Y-90 PET/CT images were compared, revealing a high correlation coefficient (0.93) between non-tumoral tissue dose values, which is also consistent with the findings of our study [15]. In the same study, higher correlation coefficients were found for HCC lesions (0.82 for glass microspheres and 0.82 for resin microspheres); however, this value was found to be 0.52 for metastatic lesions. Considering that most lesions in our study were metastatic malignancies of the liver, our findings could be interpreted in line with the results of the mentioned study. In the same study, in addition to the mean dose, V70 and D70 values were also found to be consistent between pre-treatment SPECT and post-treatment PET. In another study on this topic by Kafrouni M. et al., involving 23 patients diagnosed with HCC, a high correlation was found between pre- and post-treatment images. Additionally, they reported that having the same catheter position and the same distance from the catheter position to the arterial bifurcation increased the concordance between the two studies [24]. Additionally in a study of Richetta E. et al., comparing Tc-99m-SPECT and Y-90 PET/CT in patients treated with resin microspheres, a strong correlation was observed between dosimetry performed using both imaging methods [12]. Previous studies in the literature have mainly utilized PET/CT as the imaging method for Y-90 microspheres. In this context, high-quality diagnostic imaging with the CT component of the PET/CT is possible with the usage of intravenous contrast agents and higher mAs values, however, in routine practice, PET/CT imaging is usually performed with the low dose setting. This situation coupled with the possible respiratory artifacts may complicate the segmentation of the liver structures. However, integrated PET/MRI systems may have a potential advantage with the combination of the respiratory-gating and high soft tissue contrast of the MRI. This might provide more accurate segmentation and dosimetry for the Y-90 PET images.

There are several possible limitations of the study. First, the design of this study mostly includes retrospective patients, and the patient group was heterogenous and included several different diagnoses. Secondly, while the study included patients treated in a unilobar fashion, the behavior of microspheres could be significantly affected when treatment is applied in a segmental approach. This might be due to factors such as possible saturation of the vascular bed, especially with resin microspheres. Therefore, a specific prospective study in this context may be needed to investigate differences between unilobar and segmental approaches. Finally, we have not analyzed and compared the metrics such as BED and EUD between both imaging modalities, which may limit the findings in our study.

## Conclusions

In conclusion, both our study and the literature suggest that Tc-99m-MAA SPECT/CT can reliably predict normal liver tissue absorbed dose, maintaining its importance in treatment planning. By predicting the dose absorbed by normal liver tissue, a crucial factor for treatment planning, it allows for the delivery of the highest possible dose to tumor tissue while preserving normal liver tissue. In this context, Tc-99m-MAA SPECT/CT is likely to continue to play a role in treatment planning. However, in prediction of tumor-absorbed radiation dose, predictive value of Tc-99m-MAA SPECT/CT was greatly diminished, and lower correlation detected with Y-90 PET/MRI-derived data. Additionally, several differences were observed in dose-volume histograms calculated with Tc-99m-MAA SPECT/CT and Y-90 PET/MRI. While Y-90 PET/MRI-derived data had greater D10 values compared to Tc-99m-MAA SPECT/CT, lower D90 values were observed with Y-90 PET/MRI.

## Data Availability

The datasets analyzed in this study are available upon reasonable request.
